# Aberrant Levels of Hematopoietic/Neuronal Growth and Differentiation Factors in Euthyroid Women at Risk for Autoimmune Thyroid Disease

**DOI:** 10.1371/journal.pone.0153892

**Published:** 2016-04-19

**Authors:** Elske T. Massolt, Grigoris Effraimidis, Tim I. M. Korevaar, Wilmar M. Wiersinga, W. Edward Visser, Robin P. Peeters, Hemmo A. Drexhage

**Affiliations:** 1 Division of Endocrinology, Department of Internal Medicine, Erasmus MC, 3000 CA, Rotterdam, The Netherlands; 2 Rotterdam Thyroid Center, Department of Internal Medicine, Erasmus MC, 3000 CA, Rotterdam, The Netherlands; 3 Internal Medicine Department, Endocrinology Section, Nykøbing Falster Hospital, Fjordvej 15, 4800, Nykøbing Falster, Denmark; 4 Academical Medical Center, University of Amsterdam, 1105 AZ, Amsterdam, The Netherlands; 5 Department of Immunology, Erasmus MC, 3000 CA, Rotterdam, The Netherlands; University of Pécs Medical School, HUNGARY

## Abstract

**Background:**

Subjects at risk for major mood disorders have a higher risk to develop autoimmune thyroid disease (AITD) and vice-versa, implying a shared pathogenesis. In mood disorder patients, an abnormal profile of hematopoietic/neuronal growth factors is observed, suggesting that growth/differentiation abnormalities of these cell lineages may predispose to mood disorders. The first objective of our study was to investigate whether an aberrant profile of these hematopoietic/neuronal growth factors is also detectable in subjects at risk for AITD. A second objective was to study the inter relationship of these factors with previously determined and published growth factors/cytokines in the same subjects.

**Methods:**

We studied 64 TPO-Ab-negative females with at least 1 first- or second-degree relative with AITD, 32 of whom did and 32 who did not seroconvert to TPO-Ab positivity in 5-year follow-up. Subjects were compared with 32 healthy controls (HCs). We measured serum levels of brain-derived neurotrophic factor (BDNF), Stem Cell Factor (SCF), Insulin-like Growth Factor-Binding Protein 2 (IGFBP-2), Epidermal Growth Factor (EGF) and IL-7 at baseline.

**Results:**

BDNF was significantly lower (8.2 vs 18.9 ng/ml, *P*<0.001), while EGF (506.9 vs 307.6 pg/ml, *P* = 0.003) and IGFBP-2 (388.3 vs 188.5 ng/ml, *P* = 0.028) were significantly higher in relatives than in HCs. Relatives who seroconverted in the next 5 years had significantly higher levels of SCF than non-seroconverters (26.5 vs 16.7 pg/ml, *P* = 0.017). In a cluster analysis with the previously published growth factors/cytokines SCF clustered together with IL-1β, IL-6 and CCL-3, of which high levels also preceded seroconversion.

**Conclusion:**

Relatives of AITD patients show aberrant serum levels of 4 hematopoietic/neuronal growth factors similar to the aberrancies found in mood disorder patients, suggesting that shared growth and differentiation defects in both the hematopoietic and neuronal system may underlie thyroid autoimmunity and mood disorders. A distinct pattern of four inter correlating immune factors in the relatives preceded TPO-Ab seroconversion in the next 5 years.

## Introduction

Autoimmune hypothyroidism is characterized by a combination of clinical features, elevated serum TSH with reduced free T4 (FT4) levels, the presence of serum antibodies against thyroid antigens, and reduced echogenicity of the thyroid sonogram [1]. It is the most common organ-specific autoimmune disorder with an estimated prevalence of 2%, with a higher prevalence in women and depending on iodine intake [2–5]. Thyroid peroxidase (TPO) is the major autoantigen and TPO antibodies (TPO-Abs) are present in almost all patients with autoimmune hypothyroidism [6] and precede the clinical phase of autoimmune hypothyroidism by many years. Subclinical autoimmune hypothyroidism (the presence of TPO-Abs with raised TSH and normal FT4 levels) is even more prevalent and affects about 9% of the population [2, 5]. In the Whickham follow-up study, women with TPO-Abs had an eight-fold higher risk of developing clinically overt hypothyroidism over 20 years than did antibody-negative women [7]. In our own studies on the Amsterdam AITD cohort (euthyroid females with at least one first or second degree relative with a documented autoimmune hyper- or hypothyroidism) TPO-Ab positivity at the start of the study also represented a higher risk to develop overt hypothyroidism in a follow-up of 5 years [8, 9]. In addition, there was a higher conversion rate from TPO-Abs negativity to positivity, showing a familial proneness for thyroid autoimmune reactivity [9, 10].

In another previous study on this cohort, we tested the hypothesis that serum levels of factors related to thyroid growth and connective tissue abnormalities (Platelet-Derived Growth Factor (PDGF)-BB, Fibronectin, Metalloproteinase (MMP)-13), to the early accumulation of immune cells in the thyroid (soluble Vascular Cell Adhesion Molecule (sVCAM)-1, CCL2, CCL4, Angiopoetin-1 Receptor-2 (TIE-2)), and to inflammation (IL-1β, IL-6 and CCL3) were related to this proneness for thyroid autoimmunity in the relatives [11]. We therefore studied these factors in the serum of 64 TPO-Ab negative euthyroid relatives, 32 of whom did and 32 of whom did not seroconvert to TPO-Abs positivity in 5 year follow-up. The relatives were compared with 32 healthy controls. We found that both seroconverting and non-seroconverting relatives showed an up regulation of Fibronectin and a down regulation of PDGF-BB, CCL2, CCL4, sVCAM-1, TIE-2 and MMP-13. The relatives who later seroconverted (seroconverters, SCs) differed from those who did not seroconvert (non-seroconverters, NSCs) by a significant up regulation of pro-inflammatory compounds, such as IL-1β, IL-6 and CCL3. We concluded that euthyroid females within AITD families show a characteristic pattern of abnormalities in serum levels of growth factors, chemokines, adhesion molecules and cytokines, suggesting an already compromised thyroid-immune system interaction in the euthyroid family members. Also, pre-seroconversion stages might be predicted using serum analytes pointing to a higher inflammatory state.

Autoimmune hypothyroidism is commonly accompanied by depressive symptoms. A large epidemiological Danish nationwide, prospective cohort study showed that various autoimmune diseases including AITD, are associated with subsequent lifetime mood disorder diagnosis (e.g. bipolar affective disorder, unipolar depression, psychotic depression and other remaining mood disorders) [12]. In hypothyroid patients the lack of thyroid hormone in the brain is likely an important determinant for these mood disturbances [13]. However, a deficit of thyroid hormone may not be the only cause, as even subjects with TPO-Abs with normal thyroid function have a higher risk to develop anxiety disorders and mood disorders [14]. Also offspring of patients with a bipolar affective disorder have a higher prevalence of TPO-Abs, even if they are not affected by the psychiatric disorder [15, 16]. In addition, a higher prevalence of TPO Abs and autoimmune hypothyroidism has been reported in patients with bipolar affective disorder, irrespective of the usage of lithium [17, 18]. Taken together, these associations might imply a shared immune pathogenesis for both AITD and mood disorders.

We therefore additionally determined, in the sera used in the previous study, 5 growth and differentiation factors that have *repeatedly* been shown to be abnormally expressed in the circulation of mood disorder patients and that are capable of influencing both immune and/or neuronal cell growth, i.e. SCF, IGFBP-2, EGF, BDNF and IL-7 [19–23]. In addition we studied the inter relationship of these factors with the previously determined factors using a cluster analysis to study patterns of TPO-Ab seroconversion [11].

## Subjects and Methods

### Subjects

The Amsterdam AITD cohort has previously been described in detail [8]. In the present study, we studied serum levels of several hematopoietic/neuronal growth factors in the subjects. In addition we assessed the association with TPO-Ab seroconversion like we previously did in the study of Beumer *et al* [11]. Therefore, 32 euthyroid subjects were selected who were TPO-Ab and Tg-Ab negative at baseline but developed TPO-Abs during follow-up without developing an abnormal TSH. Each selected SC was matched with an euthyroid subject who was TPO-Ab and Tg-Ab negative at baseline and did not develop TPO-Abs (non-seroconverter) up to the time at which the SC to whom they were matched had received her endpoint. SCs and NSCs were matched for age, current smoking, current estrogen use, and duration of follow-up.

As a control group, we selected 32 female subjects from an ongoing program for delineating reference values of endocrine function tests that were in self-proclaimed good health, were not using chronic medication (except for oral contraceptives), had no family or personal history of thyroid disease, and had normal TSH and no thyroid antibodies. Blood samples were collected over the same period of time as those of the Amsterdam AITD cohort and were processed in the same manner.

All subjects gave informed written consent and the Medical Ethics Committee of the Academic Medical Center in Amsterdam and the Medical Ethics Committee of Erasmus Medical Center in Rotterdam approved the study.

### TSH, FT4 and TPO-Ab determinations

Serum samples were stored at −20°C until determination of the study parameters. Serum TSH and FT4 were measured using time-resolved fluoroimmunoassay (Delphia, Turku, Finland). Reference values are 0.4–5.7 mU/L for TSH and 9.3–20.1 pmol/L for FT4. TPO-Abs and Tg-Abs were measured by chemiluminescence immunoassays (LUMI test anti-TPO and LUMI test anti-thyroglobulin, respectively; Brahms, Berlin, Germany). Improved versions of both assays became available during follow-up: the detection limits of these new assays were 30 kU/L for TPO-Abs and 20 kU/L for Tg-Abs. The TPO-Ab concentrations obtained with the old assay were multiplied by a factor 0.72 to obtain comparative values in the new assay. TPO-Ab and Tg-Ab concentrations were considered to be positive at values greater than 100 kU/L.

### Serum growth factors

We studied a panel of five growth factors at baseline: SCF, IGFBP-2, EGF, BDNF and IL-7. Serum concentrations were measured using the bead-based Luminex system. These multiplexed sandwich immunoassays were developed from commercially available capture and detection antibodies (R&D systems) and standard proteins, validated and approved at Myriad-EDI-GmbH (Reutlingen, Germany) according to methods described previously [24]. Subject and healthy control samples were run singular. Assays were measured on either the Luminex FlexMap-3D or Luminex 200 system. Results are expressed as picograms per ml or nanograms per ml.

EGF, IL-7, SCF, BDNF were measureable in all samples. For IGFBP-2 6.6% of the values were above the detection limit. Values above the detection limit were set to the highest value observed (IGFBP-2: 19666.5 ng/ml).

### Statistics

Test assumptions were checked by plotting of the data and depending on the distribution pattern, parametric (Student’s T-test) or nonparametric group comparisons (Mann-Whitney U test) were used for unadjusted group comparisons. All analyses comparing HC, SC and NSC groups were subsequently adjusted, for which we used ANOVA. We adjusted for smoking, the usage of oral contraceptives, age, BMI and FT4 levels. Because SCs and NSCs were matched for age, smoking and estrogen use we did not adjust for these factors when comparing SCs and NSCs. If necessary we transformed dependent variables by the natural logarithm. In order to improve the interpretability of the group estimates, data are expressed as median with 95% confidence intervals which were calculated using a bootstrap procedure with 1000 draws. For IGFBP-2, residuals of the regression analyses remained non-normal after transformation due to outliers (n = 8), however, we can reliably report the outcomes of the regression analyses as these were in line with unadjusted non-parametric results and also remained similar after exclusions of the outliers.

In addition a dendrogram was constructed by SPSS using hierarchical cluster analysis of the serum analytes using the between-groups linkage method. For this analysis, we selected analytes from the previous study that were significantly different between healthy controls and subjects or between NSCs and SCs (S1 Table) and were part of a cluster in that study, and combined them with the levels of growth and differentiation factors assessed in the current study [11]. The associations between TSH, FT4 and the serum growth factors were analyzed by linear regression analyses. Level of significance was set at *P*<0.05 (2 tailed). Statistical analysis was performed using SPSS Statistics for Windows, version 21 (IBM Corp., Armonk, NY, USA).

## Results

As a result of the matching procedure, SCs and NSCs were not different regarding age, current smoking behavior, Body Mass Index (BMI), current estrogen use and TSH or FT4 levels (Table 1). None of the subjects were using chronic medication. TSH levels were not associated with any of the growth and differentiation factors (data not shown). FT4 levels were positively associated with IGFBP-2 levels (β±SE 0.16 ±0.075; *P* = 0.037) and we observed a non-significant trend with SCF (β±SE 1.2 ±0.68; *P* = 0.069). FT4 was not associated with the other growth factors.

**Table 1:** Baseline characteristics.

							*P-values*		
	Controls		SC		NSC		SC vs C	NSC vs C	NSC vs SC
**Number of subjects**	30		30		31				
**Age, mean (range)**	35.2	(21–61)	33.3	(18–61)	33.5	(19–62)	0.5	0.56	0.93
**BMI, mean (range)**	22.7	(18–33)	24.1	(19–41)	24.2	(19–42)	0.22	0.17	0.95
**Current smoking, %**	12	(40%)	14	(46%)	14	(45%)	0.61	0.69	0.9
**Current estrogen use, %**	5	(17%)	11	(37%)	12	(38%)	0.055	0.083	0.87
**TSH, median (95% CI)**	1.3	(1.1–1.7)	1.4	(1.3–1.7)	1.2	(1–1.5)	0.36	0.66	0.13
**FT4, median (95% CI)**	13.2	(12.9–14.6)	12.8	(12.4–13.5)	13.7	(13–14.2)	0.53	0.60	0.25

Characteristics of healthy controls (C) and relatives of AITD patients grouped for TPO antibody conversion during follow-up (seroconverters (SC) and non seroconverters (NSC)). Due to a lack of serum, the total number of subjects per group is not equal to the original 32.

### Subjects versus healthy controls

Table 2 shows the median levels of the 5 tested growth and differentiation factors in the healthy controls and in the subjects. IGFBP-2 levels were significantly higher in the relatives than in the healthy controls (*P* = 0.028). Serum levels of EGF were also significantly higher in the relatives than in the healthy controls (*P* = 0.003). Serum levels of BDNF were significantly lower in the relatives than in the healthy controls (*P*<0.001). Serum levels of SCF and IL-7 were not statistically different between relatives and healthy controls.

**Table 2:** Comparison of growth and differentiation factors between healthy controls and subjects.

	Controls	Subjects	*P*-value	Adjusted *P*-value
**EGF (pg/ml)**	307.6 (110–409)	506.9 (428–612)	0.001	0.003
**BDNF (ng/ml)**	18.9 (14.6–22.7)	8.2 (7.3–9.3)	<0.001	<0.001
**IGFBP-2 (ng/ml)**	177.8 (142–235)	252.5 (177–351)	0.073	0.028
**SCF (pg/ml)**	22.6 (17.4–26.4)	22.6 (16.7–27.1)	0.58	0.70
**IL-7 (pg/ml)**	4.0 (2.8–5.1)	3.7 (3.0–3.8)	0.18	0.26

Median levels (95% CI) of EGF, BDNF, IGFBP-2, SCF and IL-7 in healthy controls and in subjects overall. Adjusted P-values are adjusted for age, BMI, smoking, oral contraceptive usage and FT4 levels.

### Seroconverters versus non-seroconverters

Table 3 shows that SC subjects had significantly higher levels of SCF than NSC subjects (*P* = 0.017). Serum levels of SCF were not statistically different between SCs and healthy controls and between NSCs and healthy controls (*P* = 0.49 and *P* = 0.26, respectively). The serum levels of IGFBP-2, EGF, BDNF and IL-7 were not significantly different between SCs and NSCs. Serum levels of EGF were significantly higher in SCs and in NSCs than in healthy controls (*P* = 0.017 and *P* = 0.011, respectively). Serum levels of BDNF were significantly lower in SCs and in NSCs than in healthy controls (*P*<0.001 in both groups). A non-significant trend towards lower levels of IL-7 in the SCs than in controls was observed (*P* = 0.076). IGFBP-2 levels were higher in both groups of subjects than in healthy controls which was significant in the NSCs (*P* = 0.019).

**Table 3:** Serum levels of growth and differentiation factors in healthy controls (C), Seroconverting (SC) and Non-Seroconverting (NSC) family members.

				Adjusted *P*-value		
	Controls	SC	NSC	SC vs C	NSC vs C	SC vs NSC
**EGF (pg/ml)**	307.6 (110–409)	564.2 (411–803)	470.6 (355–612)	0.017	0.011	0.79
**BDNF (ng/ml)**	18.9 (14.6–22.7)	8.7 (5.1–10.8)	8.1(6.9–9.3)	<0.001	<0.001	0.45
**IGFBP-2(ng/ml)**	177.8 (142–235)	233.2 (153–376)	252.5 (164–390)	0.16	0.019	0.31
**SCF (pg/ml)**	22.6 (17.4–26.4)	26.5 (22.5–31.9)	16.7 (12.3–22.8)	0.49	0.26	0.017
**IL-7 (pg/ml)**	4.0 (2.8–5.1)	3.4 (2.7–3.8)	3.7 (3.0–4.0)	0.076	0.55	0.25

Median levels (95% CI) of EGF, BDNF, IGFBP-2, SCF and IL-7.

### Cluster analysis

**Fig 1:**
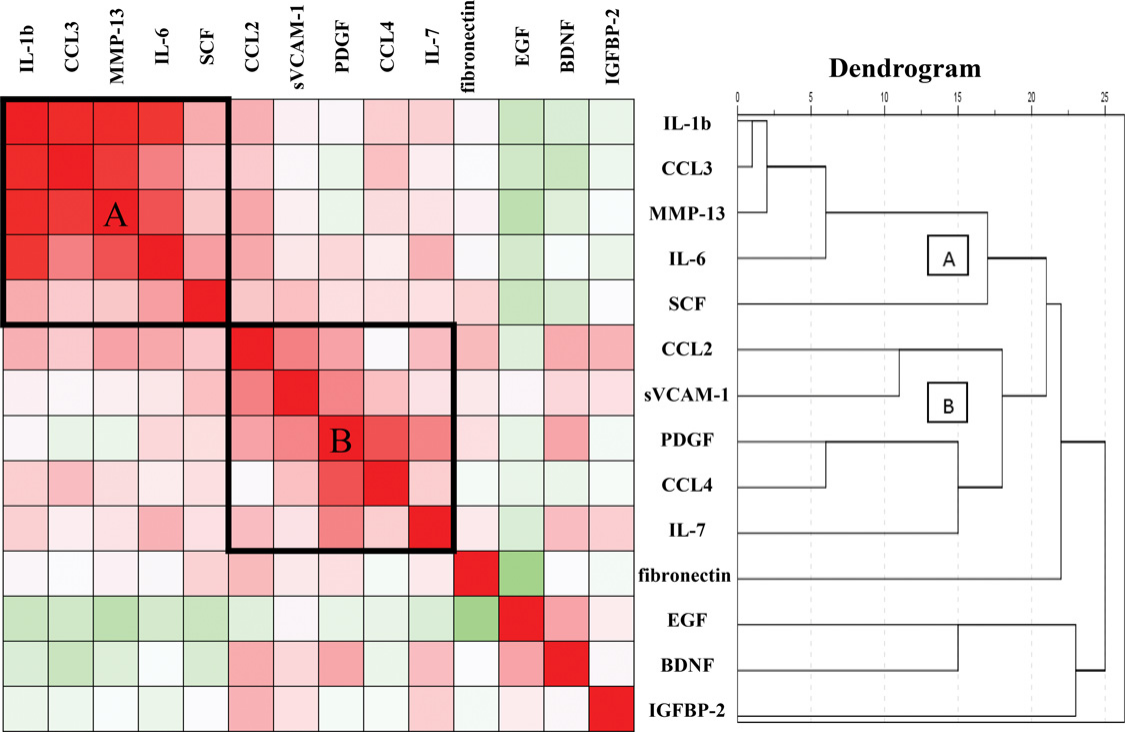
Cluster analysis. Heat map of hierarchical cluster analysis of the serum levels of cytokines, chemokines, growth factors and tissue remodeling factors in the relatives. Color-coded correlation matrix illustrates Pearson’s correlation coefficients. Significant positive correlations are given in the red scale (darkest red are correlation coefficients > 0.50), and significant negative correlations are given in the (dark) green scale. Lighter fields are not significant. In addition, a dendrogram is presented as a result of the hierarchical clustering. A indicates cluster A and B indicates cluster B.

Taking the analytes which we have previously determined also into account we found in the cluster analysis two mutually correlating clusters of analytes: one stronger inter correlating cluster A and a weaker inter correlating cluster B (Fig 1) [11]. Cluster A contained the inflammatory cytokines/chemokines IL-1β, CCL3 and IL-6, the connective tissue modulator MMP-13 and the hematopoietic/neuronal growth and differentiation factor SCF, while cluster B contained the pro-inflammatory chemokines CCL2 and CCL4, the endothelial adhesion molecule sVCAM-1, PDGF-BB, and the T cell and NK cell growth factor IL-7. The heat map shows that the growth factors IGFBP-2, EGF and BDNF barely correlated to each other and to the other analytes.

Regarding the pattern of expression in the two study groups (SCs and NSCs) three patterns of reactivity could be detected (S1 Table and Table 4). There were factors, which were higher in the serum of both SCs and NSCs than in controls, such as the growth factors IGFBP-2, EGF and the repair factor Fibronectin. There were also factors that were lower in the serum of both SCs and NSCs than in controls, such as CCL4, CCL2, sVCAM-1, BDNF and PDGF-BB. Finally, there were factors that were higher in the SCs than in the NSCs such as IL1-β, IL-6, CCL3 and SCF (S1 Table and Table 4).

**Table 4:** Patterns of expression levels of cytokines, chemokines and growth and differentiation factors, assessed in the previous study of Beumer *et al*. and in the current study [7].

	NSC vs HC	SC vs NSC	SC vs HC
**Pattern 1**			
Fibronectin (μg/ml)	↑↑	≈	↑↑
IGFBP-2 (ng/ml)	↑↑	≈	≈
EGF (pg/ml)	↑↑	≈	↑↑
**Pattern 2**			
CCL4 (pg/ml)	↓↓	≈	↓↓
MMP-13 (ng/ml)	↓↓	≈	↓↓
CCL2 (pg/ml)	↓↓	≈	≈
sVCAM-1 (μg/ml)	↓↓	≈	↓↓
PDGF-BB (pg/ml)	↓↓	≈	↓↓
BDNF (ng/ml)	↓↓	≈	↓↓
**Pattern 3**			
IL-1β (pg/ml)	↓↓	↑↑	≈
IL-6 (pg/ml)	↓↓	↑↑	≈
CCL3 (pg/ml)	↓↓	↑↑	≈
SCF (pg/ml)	≈	↑↑	≈
**Other**			
IL-7 (pg/ml)	≈	≈	≈

Patterns of expression levels in serum compounds between seroconverters (SC) and non-seroconverters (NSC) and healthy controls (HC). ↑↑ and ↓↓ indicates significantly higher or lower serum levels respectively, and ≈ indicates that serum levels are not significantly different (S1 Table).

## Discussion

The present study shows that euthyroid females, who are relatives of AITD patients and at risk of developing AITD, have an aberrant serum level of 4 of the 5 tested hematopoietic/neuronal growth and differentiation factors, i.e. of BDNF, IGFBP-2, EGF and SCF. BDNF levels were significantly lower and IGFBP-2 and EGF higher expressed in sera of the relatives of the AITD patients (in both SCs and NSCs) than in healthy controls. IL-7 levels were normal. We also found in the healthy relatives, who converted in the following 5 years to TPO-Ab positivity, significantly higher serum levels of SCF than in relatives who did not.

It is of note that the 5 studied factors have been highlighted as serum biomarkers for major mood disorders in several studies [19–23] and are involved in neurogenesis, neuroprotection and hematopoietic differentiation [25–29]. This is in particular known for BDNF. Neurotrophic factors, like BDNF, play an important role in neuronal plasticity, modulating not only axonal and dendritic growth and remodeling, but also neurotransmitter release and synapse formation [30, 31]. Neuronal plasticity is a complex process which is illustrated by the complex interaction between the neurotrophic factors and its receptors. The cellular actions of BDNF, for example, are mediated through TrkB (tyrosine kinase receptor) and by p75 neurotrophin receptor (p75^NTR^) [32]. Binding of dimeric BDNF causes dimerization of TrkB receptor and autophosphorylation of intracellular tyrosine residues. Activation of TrkB receptor leads to signaling cascades involving activation of Ras/ERK pathway, phosphatidylinositol 3-kinase (PI3K) and Phospholipase Cγ [33]. The Ras pathway regulates neuronal survival and differentiation through downstream signaling that includes c-RAF/B-Raf/ERK1/ERK2. Brunoni *et al*. showed in a meta-analysis that BDNF levels were decreased in patients with a major depressive disorder and were also associated with clinical changes in depression [34].

This study also extends our previous study and shows that the here reported 5 growth factors and the previously reported 9 growth factor/cytokines form three patterns of reactivity in the relatives of AITD patients when compared to healthy subjects and depending on TPO-Ab seroconversion within the next 5 years [11]. This study and the previous one therefore underscore the widespread changes in immune-neuro-endocrine molecular networks that apparently precede the appearance of TPO-Abs, which opens avenues for developing assays for the detection of individuals at risk for thyroid autoimmunity.

Combining this study with the previous one we found factors which were raised in the relatives, irrespective of later seroconversion, such as the growth factors IGFBP-2, EGF and the repair factor Fibronectin. None of these factors inter correlated. There were also factors that were reduced in the serum of the relatives irrespective of later seroconversion, such as the chemokines CCL4, CCL2, the adhesion molecule sVCAM-1, and the growth factors BDNF and PDGF-BB. Many of these factors inter correlated in cluster B in the dendrogram constructed in this article. Taken these two patterns of reactivity together, the serum aberrancies suggest another state of growth regulation of multiple cell lines (including neuronal and hematopoietic cells) and another state of leukocyte migration in relatives of AITD patients.

Finally, there were factors that were higher in the SCs than in the NSCs such as IL1-β, IL-6, CCL3 and SCF, while often being lower in the NSCs than in controls. These factors correlated to each other in cluster A in the dendrogram. We assume that the generally low expression in NSCs in cluster A reflects an immune suppressive state preventing autoimmunity, while a rise of these pro-inflammatory compounds precedes a conversion to TPO-Ab positivity and thus may reflect a very early stage of thyroid auto reactivity. In recent studies, the importance of T helper 1 lymphocytes in the induction of such auto-inflammatory state has been shown [35]. A further investigation of cytokines and chemokines reflecting the higher state of the T helper 1 system (such as IL-12, IFN-γ and CXCL-10) in relatives of AITD index cases deserves further attention [36, 37].

A limitation of our study is the relatively small sample size. Also, because this is an explorative study which focused on 5 non-random selected analytes, we did neither take type I errors into account nor applied a correction for multiple testing. Our study is also limited by the fact that we did not assess the association between the levels of these growth and differentiation factors and the mood state of the relatives (sampling of the sera occurred 15 years ago, at the time we were unaware of the link between AITD and depression). Next, we used a BDNF antibody in our immunoassay, which was developed to measure mature BDNF. It is now known that there are other assays and antibodies on the market that also measure pro-BDNF [38]. It has been reported, however, that in particular mature BDNF is important in major depression [39] although this needs exploration. Furthermore, also other factors important in neuronal growth and differentiation should be taken into account, such as glial-cell-line-derived neurotrophic factor (GDNF). GDNF signals through a multicomponent receptor complex comprising the Ret proto-oncogene (RET) tyrosine kinase and the GDNF family receptor (GFR)α [40]. Following GDNF binding in the presence of co-receptor GFRα, RET becomes dimerized and tyrosine phosphorylated and triggers different pathways (Ras-MAPK, PI3K-Akt). Finally, in the paragraph above, we have highlighted that other immune factors which are linked to the induction of thyroid autoimmunity (such as T helper 1 related factors) should be explored. It is also evident that our studies need confirmation and expansion in larger families and follow-up studies, taking many more neuronal, endocrine and immune factors into consideration to unravel the changes in immune-neuro-endocrine molecular networks that precede and probably underlie the development of AITD and mood disorders.

Since a large number of autoimmune hypothyroid patients have a diminished cognitive and psychological function despite adequate levothyroxine replacement therapy, another next step could be to measure the growth and differentiation factors in these patients and to assess the association with these symptoms.

We conclude that subjects at risk for AITD show changes in growth and differentiation factors in serum, which are both active as neuronal and hematopoietic growth and differentiation factors and are abnormally expressed in patients with mood disorders. This suggests that shared growth and differentiation defects in both the hematopoietic and neuronal system may underlie both thyroid autoimmunity and mood disorders.

## Supporting Information

S1 Table Serum levels of cytokines, chemokines and growth factors of healthy controls (HC), Non-Seroconverting (NSC) and Seroconverting (SC) relatives assessed in the previous study and in the current study, grouped according to patterns of expression. (DOCX)
