# Supplementary material for: Aberrant Levels of Hematopoietic/Neuronal Growth and Differentiation Factors in Euthyroid Women at Risk for Autoimmune Thyroid Disease
Source: PLoS One. 2016 Apr 19;11(4):e0153892. doi: 10.1371/journal.pone.0153892 (PMC4836766; doi:10.1371/journal.pone.0153892)
Supplement: S1 Table — (DOCX) [file pone.0153892.s001.docx]

|  |  | **S1 Table.** Serum levels of cytokines, chemokines and growth factors of healthy controls (HC), Non-Seroconverting (NSC) and  Seroconverting (SC) relatives assessed in the previous study and in the current study, grouped according to patterns of expression. | | | | | | | | | | | | |
| --- | --- | --- | --- | --- | --- | --- | --- | --- | --- | --- | --- | --- | --- | --- |
|  | | | Pattern | Cluster | HC | | NSC | | SC | | NSC vs HC | SC vs NSC | SC vs HC |  |
|  | | |  |  | *M* | *SE* | *M* | *SE* | *M* | *SE* | *P value* | *P value* | *P value* |  |
| Fibronectin (µg/ml) | | | 1 |  | 367.1 | (47.5) | 646.2 | (33.6) | 590.6 | (28.4) | **<0.001** | 0.21 | **<0.001** |  |
| IGFBP-2 (ng/ml) | | | 1 |  | 848.1 | (649.2) | 3410.4 | (1302) | 1552.1 | (899.4) | **0.019** | 0.20 | 0.16 |  |
| EGF (pg/ml) | | | 1 |  | 314.6 | (43.4) | 598.1 | (70.1) | 637.0 | (86.1) | **0.011** | 0.80 | **0.017** |  |
| CCL4 (pg/ml) | | | 2 | B | 77.8 | (25.5) | 23.4 | (18.6) | 6.9 | (5.7) | **<0.001** | 0.13 | **<0.001** |  |
| CCL2 (pg/ml) | | | 2 | B | 474.4 | (31.7) | 191.7 | (28.1) | 166.6 | (24.4) | **<0.001** | 0.32 | 0.271 |  |
| sVCAM-1 (ng/ml) | | | 2 | B | 1246.8 | (98.9) | 481.1 | (39.7) | 491.0 | (37.1) | **<0.001** | 0.93 | **<0.001** |  |
| PDGF-BB (pg/ml) | | | 2 | B | 2470 | (364.5) | 1145.7 | (72.0) | 1017.7 | (47.9) | **<0.001** | 0.12 | **<0.001** |  |
| BDNF (ng/ml) | | | 2 |  | 17.9 | (1.7) | 8.7 | (0.7) | 7.9 | (0.7) | **<0.001** | 0.45 | **<0.001** |  |
| MMP-13 (ng/ml) | | | 2 | A | 2.2 | (0.6) | 0.62 | (0.1) | 1.1 | (0.3) | **<0.001** | 0.27 | **0.001** |  |
| IL-1B (pg/ml) | | | 3 | A | 269.3 | (89.1) | 72.2 | (48.1) | 564.2 | (318.8) | 0.004 | **0.012** | 0.82 |  |
| IL-6 (pg/ml) | | | 3 | A | 10.5 | (5.7) | 1.8 | (0.7) | 20.3 | (13.5) | 0.011 | **0.041** | 0.47 |  |
| CCL3 (pg/ml) | | | 3 | A | 631.6 | (177.6) | 209.9 | (67.1) | 1672.6 | (1036.6) | 0.012 | **0.009** | 0.74 |  |
| SCF (pg/ml) | | | 3 | A | 24.9 | (2.3) | 20.0 | (2.3) | 26.7 | (2.3) | 0.26 | **0.017** | 0.49 |  |
| IL-7 (pg/ml) | | |  |  | 5.3 | (1.0) | 3.9 | (0.3) | 3.3 | (0.3) | 0.23 | 0.55 | 0.076 |  |

The mean (M) and standard error (SE) are given. For parameters with a non-normal distribution non-parametric statistical testing was applied.
